# Psychopathic personality traits are associated with experimentally induced approach and appraisal of fear-evoking stimuli indicating fear enjoyment

**DOI:** 10.1038/s41598-025-91652-2

**Published:** 2025-03-13

**Authors:** Sabrina Schneider, Miriam J. Hofmann, Andreas Mokros

**Affiliations:** Faculty of Psychology, FernUniversität in Hagen, 58097 Hagen, Germany

**Keywords:** Psychopathy, Fear enjoyment hypothesis, Approach-avoidance task, Human behaviour, Signs and symptoms

## Abstract

The extent to which deficits in the perception and experience of fear contribute to psychopathic symptoms is an ongoing matter of debate. Traditional theories emphasize diminished threat processing as the core fear deficit in psychopathic individuals, whereas recent approaches, such as the fear enjoyment hypothesis (FEH), propose that anomalies in the subjective experience of fear are related to interpersonal-affective psychopathic traits. In order to test predictions of the FEH, we conducted an online experimental study to examine whether approach and avoidance tendencies in response to fear-eliciting stimuli are related to specific psychopathic traits (interpersonal-affective symptoms and boldness). We expected these traits to be associated with faster approach towards and slower avoidance of fear-evoking images in an approach-avoidance task (AAT). In a community-based sample of 211 individuals (69% female), self-reported interpersonal-affective traits predicted more positive appraisal ratings of and a faster approach towards fear-evoking stimuli in the AAT paradigm. The link between psychopathic traits and faster approach towards fear images could not be explained by traits related to impulsiveness or impaired inhibitory control. The present findings shed light on the subjective appraisal of fear-evoking situations by individuals with elevated psychopathic traits. Implications on etiological models of psychopathy are discussed.

## Introduction

Fear deficits have long been associated with the notion of the psychopathic personality. Some etiological theories, e.g.,^1–3^ attribute psychopathy-associated traits and behaviors, including high impulsivity, a lack of guilt, and antisocial tendencies, to fundamental deficits in emotion processing, which are typically assumed to involve the amygdala^4,5^. In addition to these theories which postulate specific assumptions regarding the development of psychopathic personality traits, altered fear processing is also included in different conceptual models that focus on the assessment and phenomenological descriptions of psychopathic personality characteristics, e.g.,^6,7^. Hence, fear anomalies seem to prevail across different models and theories that provide a framework for the clinical assessment and definition of psychopathy.

### Fear deficits in common psychopathy models

According to Hervey M. Cleckley^8,9^, psychopaths are unable to develop morality through adequate socialization due to an innate attenuated emotion sensitivity. Fearlessness, however, was never considered as a major diagnostic marker in Cleckley’s clinical description of psychopathy (see also Neumann et al.^10^). Rather, Cleckley noted the absence of problematic anxiety, rather than a problematically low level of anxiousness, in highly psychopathic individuals^9^. In another early attempt at organizing psychopathic symptoms, Karpman^1^ grouped psychopathy-related traits and behavior prevalent in psychiatric settings into two broad categories. Thereby, he distinguished ideopathic or primary psychopathy from symptomatic or secondary psychopathy. The former was meant to describe individuals with a heritable affective defect resulting in a fearless and predatory character; the latter (i.e., the symptomatic/secondary variant) referred to “cases of psychoses and neuroses that have a strong antisocial or delinquent aspect” (p. 533)^1^. Although the primary variant clearly includes fearlessness or low levels of anxiety as diagnostic markers, the second is characterized by increased levels of anxiety and neuroticism. Hare^11^, whose assumptions are strongly rooted in Cleckley’s^9^ phenomenological description of psychopathy, summarized relevant characteristics in a structural model that has been empirically confirmed in different culture and language contexts^10,12–14^. Hare postulated two higher-order factors, with Factor 1 comprising the facets Interpersonal (e.g. pathological lying) and Affective (e.g. lack of remorse) and Factor 2 including the facets Antisocial (e.g. weak behavioral control) and Lifestyle (e.g. irresponsibility). According to Hare and Neumann^15^ and Neumann et al.^10^, each PCL-R factor taps into aspects of low anxiety and fearlessness, whereby both are thought to be an integral part of psychopathy rather then representing distinct aspects that are separate from its dimensions.

Hare’s concept focuses on pathological and criminogenic traits to describe the psychopathic personality, whereas some recent models also consider adaptive traits such as fearless dominance^6^ or boldness^7^ as relevant. According to Patrick^16^, the Triarchic Psychopathy Measure (TriPM) captures the three dimensions of boldness, meanness, and disinhibition, which are thought to be based on distinct mental processes^7^. In this sense, meanness and boldness are attributed to reduced fear processing, whereas disinhibition is ascribed, among other things, to impaired inhibitory control. A predisposition to subdued fear processing is assumed to lead to a high level of boldness or meanness as a result of developmental experiences, risk factors, and (a lack of) protective factors^7^. In their temperament-based dual pathway model, Fowles and Dindo^3^ postulated at least two conditions of temperament that are relevant to the development of psychopathy. More specifically, low fear/anxiety and strong reward or approach motivation are considered to represent central risk factors for Factor 1 psychopathy symptoms. Factor 2, in turn, is supposed to be predicted by impulsivity/disinhibition, anger/aggression, and anxiety/depression. Although low fear is not regarded as the most critical prerequisite for a negative developmental trajectory, fear deficits are thought to reduce the influence of positive environmental factors in children who are at risk of developing psychopathic symptoms^3^.

The most detailed theoretical account on the contribution of fear deficits to psychopathic symptoms is presented in Lykken’s low fear hypothesis^2,17^. According to this theory, psychopathic individuals have a “below average endowment of innate fearfulness” (p. 154)^2^. Hereby, Lykken proposed a reduced response to threat in psychopathic individuals, leading to impaired fear reactions to (threatened) punishment, which would ultimately result in the avoidance of aversive or sanctioned behaviors. Instead, psychopathic individuals continue to show aversive behavior, especially in the presence of (potential) rewards.

### Empirical evidence for fear deficits in psychopathy

Initial empirical support for Lykken’s theory stems from his own seminal investigation of the association between psychopathy and fear^17^. This study was based on a classical conditioning paradigm with an electric shock as unconditioned stimulus, a buzzer serving as conditioned stimulus, and electrodermal activity (EDA) as the conditioned response (i.e., the dependent variable). Psychopathic individuals showed overall reduced EDA responses in anticipation of shock. Subsequent studies confirmed impaired fear conditioning and poor avoidance learning in psychopathic adults (e.g. Benning et al.^18^, Newman and Kosson^19^, Newman and Schmitt^20^, for a detailed overview see Hofmann et al.^21^) using a wide range of (psychophysiological) methods as measures of decreased threat reactivity, such as EDA, heart rate, or affective startle reflex modulation (for an overview see^22^). Most of the corresponding studies support Lykken’s notion that psychopathy is characterized by a diminished fear response to threat. Nevertheless, recent theoretical accounts challenged the idea that this impairment reflects a genuine emotional deficit. According to attention-based theories^23–26^, psychopathic traits evolve from an interplay of general deficits in information processing, which, in turn, lead to a reduced responsiveness to threat or fear-eliciting stimuli (as those stimuli are not processed adequately). In fact, recent research suggests that deficits in threat processing may, at least in part, be a consequence of impaired information processing in general^27^. These findings, however, do not contradict the possibility of psychopathy-related anomalies in the *subjective experience* of fear. While the initial perception of fear-inducing stimuli and environments (and, concomitantly, the psycho-physiological response) occurs at an early stage of emotional processing, the subjective fear experience occurs at a later stage and involves a conscious appraisal of the respective affect^21^. Notably, this subjective appraisal level of a fear response to (potential) threat was largely disregarded in psychopathy research so far.

### The fear enjoyment hypothesis

As mentioned above, most studies assessing fear in psychopathy were conducted in a way that examined automatic responses to threat as an indicator of an innate fear deficit, largely neglecting the subjective quality of the emotion experienced by the individual. In other studies, participants were asked to identify emotional states from facial stimuli (or similar)^28,29^, whereby a reduced recognition accuracy for fear was interpreted in terms of a fear processing deficit^28^. This approach is problematic as it mingles the recognition of fear in other individuals with one’s own personal fear response and experience. The few extant studies exclusively assessing subjective feelings in affect-laden situations (e.g., while watching emotionally arousing video clips) found an association between affective-interpersonal psychopathic traits and more positive emotional experiences when watching fear-evoking videos^30–32^. The authors of these studies concluded that individuals with pronounced psychopathic traits interpret their own emotional reactions in fear-inducing situations differently and may thus experience positive feelings in the face of threat, what became known as the *fear enjoyment hypothesis* (FEH)^31^. This idea is in line with previous research indicating similar physiological responses to fear-evoking and positive/appetitive stimuli in psychopathic individuals^33,34^. Moreover, a recent registered report provided additional empirical support of the FEH, and further showed that both self-reported fear enjoyment and the positive appraisal of fear-evoking stimuli is consistently related to Factor 1 psychopathic traits, whereas the results on putative links with boldness (as measured with the TriPM^16^) were inconclusive^32^. Thus, the FEH may be able to integrate heterogeneous findings on psychopathic fear processing and, therefore, yield a significant theoretical contribution to gaining insight into the affective processes underlying psychopathy.

### The present study

According to the FEH, fear-evoking or threat stimuli can be considered as appetitive cues for psychopathic individuals. The well-known Reinforcement Sensitivity Theory (RST)^35,36^ and its revised version (rRST)^37^ suggest a neurobiological motive system which regulates behavior in response to positive-rewarding vs. negative-punishing stimuli and situations. Hereby, the *behavioral activation system* (BAS) is sensitive to both unconditioned and conditioned rewards as well as to the cessation of punishment, thus regulating the active approach towards positive outcomes and the active avoidance of negative outcomes. The other two components, i.e., the *fight/flight/freeze system* (FFFS) and the *behavioral inhibition system* (BIS), become activated by the presence of aversive cues. The FFFS regulates immediate fear (flight or freeze) as well as aggressive (fight) responses to acute threat, whereas the BIS inhibits approach behavior and strengthens withdrawal and passive avoidance tendencies (i.e., increased anxiety and risk aversion) when both negative outcomes and reward cues are present simultaneously. Simply put, pleasant stimuli produce automatic approach tendencies, whereas negative stimuli produce automatic avoidance tendencies^38^. If threat is indeed subjectively perceived as something positive by psychopathic persons, in line with the RST, the BAS should activate behavior that fosters approach towards these stimuli. As a consequence, based on this framework the FEH predicts that individuals with higher expressions of (Factor 1) psychopathic traits would show (1) increased approach towards and (2) reduced avoidance of fear-evoking stimuli^39^. These assumptions have not been tested empirically so far. So-called *approach-avoidance tasks* (AAT; for an overview see Fricke and Vogel^40^ or Krieglmeyer et al.^41^) provide an opportunity to assess approach and avoidance tendencies in response to different types of stimuli directly. Within the AAT paradigm, participants indicate approach to or withdrawal from a stimulus presented on screen by pressing a button or using a joy-stick. The AAT combines stimulus-response (SR) compatible task conditions (i.e., participants are instructed to approach positive and avoid negative stimuli) with SR-incompatible conditions (i.e., approach negative, avoid positive stimuli). Because BAS-regulated, automatically evoked stimulus-compatible reactions (avoid negative, approach positive) need to be suppressed in the SR-incompatible condition, prolonged reaction times (RTs) for SR-incompatible as compared to SR-compatible trials typically occur, resulting in the so-called *stimulus-response compatibility effect* (SRC effect), a core parameter in AAT paradigms^41^. As individual differences shape approach-avoidance behavior^40,42,43^, the SRC effect can be regarded as an indirect measure of subjective stimulus valence: The more positive or rewarding a stimulus is for an individual, the faster the resulting approach and the slower the resulting avoidance responses (and vice versa for negatively evaluated stimuli). Against this background, we used the AAT paradigm in the present research to investigate individual differences in automatic approach and avoidance tendencies towards pleasant and fear-eliciting picture stimuli, relating these tendencies with individual differences in psychopathic personality traits. In particular, we predicted that affect-related psychopathic traits (i.e., Factor 1, boldness) would be associated with increased approach toward fear-provoking stimuli (Hypothesis 1: psychopathic threat approach) and reduced avoidance of fear-provoking stimuli (Hypothesis 2: psychopathic non-avoidance of threat).

## Results

### Manipulation checks

Before testing the hypotheses, we conducted tests to check the plausibility of our experimental data.

First, we assessed whether study participants clearly discriminated the picture stimuli used in the AAT with respect to their (un-)pleasantness. To this end, we conducted a paired *t*-test in order to statistically compare the mean valence ratings (ranging from 1: *very negative *to 7: *very positive*) obtained for the pleasant and the fear-evoking images. This comparison revealed a clear difference in stimulus valence ratings ($t(191) = 57.57, p <.001, d = 4.16$), with pleasant stimuli being rated as far more positive ($M = 5.89$, $SD = 0.61$) than the fear-evoking stimuli ($M = 1.78$, $SD = 0.55$).

Second, we examined effects of stimulus category (pleasant, fear-evoking) and stimulus-response compatibility (SR-compatible, SR-incompatible) on response latencies in the AAT. A $2 \times 2$ repeated measures analysis of variance indicated a main effect of compatibility ($F(1,201) = 32.35, p <.001$, partial $\eta ^2 =.14$), with overall faster responses in the SR-compatible ($M = 738.33, SD = 10.61$) as compared to the SR-incompatible ($M = 782.80, SD = 13.48$) AAT condition (see also Fig. 2). This particular finding replicates previous effects using this paradigm^44,45^ and attests to the internal validity of our experimental procedure. No main effect of emotion category ($F(1,201) = 0.06, p =.815$, partial $\eta ^2 =.00$) emerged, indicating that, overall, response times did not differ between fear-evoking and pleasant stimuli. Similarly, no interaction effect ($F(1,201) = 2.17, p =.142$, partial $\eta ^2 =.01$) was observed, suggesting that SRC effects did not depend on the stimulus category and were about the same for fear-evoking and pleasant pictures.

### Hypothesis tests

Table 1 displays descriptive statistics and bivariate interrelations between the core study variables.

According to the correlation analyses, faster approach toward fear-inducing stimuli was related to higher expressions in Factor 1 ($r = -.20, p =.002$). Reaction times for avoiding fear or approaching pleasant stimuli (i.e., stimulus-compatible response latencies) were, however, unrelated to psychopathic traits (see Table 1). A faster avoidance of pleasant stimuli, however, was linked with both Factor 1 ($r = -.21$, $p =.001$), in particular the Affective facet, and Factor 2 psychopathic traits ($r = -.17$, $p =.008$). A significant correlation between SRC effects for fear-evoking stimuli was only found with Factor 1 ($r = -.18$, $p =.005$). Factor 2, on the contrary, showed weak and nonsignificant relationships with both, SRC effects for fear-evoking ($r = -.15$, $p =.015$) and for joy stimuli ($r = -.14$, $p =.027$). None of the TriPM factors was related to any of the SRC effects, neither for the fear-evoking ($-.10 \le r \le -.03$, $p >.05$), nor for the joy-related stimuli ($-.12 \le r \le -.07$, $p >.05$).

In light of the absence of associations between SRC effects and any of the TriPM dimensions, follow-up multiple regression analyses of SRC effects were conducted only for the four factors of the Self-Report Psychopathy Scale-Fourth Edition (SRP 4) only. These regression analyses showed that Factor 1 had an incremental relationship with SRC effects for fear stimuli, which was independent of Factor 2 (see Table 2). As also shown in Table 2, SRC effects for pleasant stimuli were neither predicted by Factor 1 nor by Factor 2 in the multiple regression model.

Analyses of the post-experimental stimulus ratings revealed that more positive ratings of fear-evoking stimuli were associated with higher expressions of psychopathic traits, in particular Factor 1 ($r =.37, p <.001$), Factor 2 ($r =.30, p <.001$), Meanness ($r =.28, p <.001$), and Disinhibition ($r =.20, p =.004$ cf. Table 1). SRP 4 Factor 1 ($r =.23, p <.001$) and TriPM Disinhibition ($r =.20, p =.003$) further exhibited small negative correlations with subjective valence ratings of pleasant stimuli, indicating that more negative appraisals of pleasant images were related to higher expressions on those traits.

## Discussion

This study presents the first empirical test of two core assumptions of the FEH, according to which individuals with higher expressions of psychopathic traits would show (1) increased approach towards and (2) decreased avoidance of fear-evoking stimuli.

Both predictions stem from the assumption that psychopathic individuals interpret threatening situations—in which most people feel fear—as something exciting. In other words, fear-inducing stimuli are expected to have a more positive subjective valence (or hedonic quality) for highly psychopathic persons. Direct assessments of subjective stimulus valence provided preliminary support for this assumption^30–32^. The present study extends this prediction experimentally, through systematic assessments of approach and avoidance tendencies. This seems particularly compelling given that both the FEH and differences in approach/avoidance behavior can be traced back to the Reinforcement Sensitivity Theory^36,37^.

In the present study, subjective ratings of fear-evoking and pleasant picture stimuli, which were used in our AAT, were in accordance with the expected pattern: The higher self-reported psychopathic trait expressions, the more positive were the participants’ appraisals of fear-evoking picture stimuli. In line with recent findings^32^, this relationship was most prominent for SRP 4 Factor 1, i.e. interpersonal-affective psychopathic traits.

Notably—and most relevant with regard to our study aim and hypotheses—Factor 1 traits were not only associated with the direct assessment (i.e., self-reports) of stimulus valence. More decisively, the Factor 1 traits showed significant relationships with SRC effects for fear-evoking stimuli obtained in the AAT. The negative association between Factor 1 and the SRC effects for fear stimuli indicates that elevated psychopathic traits were related to a smaller discrepancy between approach and avoidance response latencies to fear-evoking stimuli. Block-wise bivariate correlations (i.e., when latencies in SR-compatible and SR-incompatible blocks are considered separately) between response latencies and Factor 1 indicated that this shift was mostly due to faster approach towards fear-evoking pictures (in the SR-incompatible condition) rather than a slower avoidance of these stimuli (in the SR-compatible condition), supporting our first hypothesis. Based on these block-wise association patterns, our second hypothesis, that increased psychopathic traits would be linked to decreased (i.e., slower) avoidance of fear-evoking stimuli, was not supported. Correlation coefficients between Factor 1 traits and latencies during the avoidance of fear (SR-compatible AAT condition) were not only non-significant, but also negative (instead of positive, as expected) and negligible in size ($r = -.11$ to $-.08$, see Table 1). Table 1 shows that psychopathic traits seemed to be related to a general decrease in response latencies (most correlations between psychopathic traits and AAT latencies had a negative value), which could be linked with psychopathy’s disinhibited/impulsive profile. To this end, the expected positive correlation between psychopathic traits and avoidance latencies in response to fear stimuli might have been confounded with the general effect of overall faster responses provided by individuals with more psychopathic traits. Although it seems reasonable to assume that a more positive appraisal of stimuli leads to a decreased tendency to avoid them, we argue that increased/facilitated approach behavior in response to such stimuli is even more indicative of their subjective pleasantness, because an approach bias is regarded as an index of strong BAS activity in general^37^ and in AAT paradigms in particular^40^. In that sense, we conclude that the results of our regression and correlation analyses essentially support the FEH.

At first sight, however, the results of the bivariate correlation analyses (cf. Table 1) could further indicate that Factor 1 had a general speed effect on stimulus-incompatible response latencies: Significant negative correlations of Factor 1 were found with both the approach towards threat *and* the avoidance of pleasure (see Table 1). Subsequent step-wise multiple regression analyses revealed, however, that the SRC effects, as the core parameter of AAT paradigms, were related to psychopathic traits only for fear-evoking, but not for the pleasant stimuli. As the SRC effect is typically interpreted in terms of implicit stimulus valence^40,43^ this finding suggests a shift on the negative-positive valence continuum towards a more positive appraisal of fear-evoking stimuli in individuals with higher levels of psychopathic traits, as mentioned above. This shift was not observed for the pleasant stimuli, as psychopathic traits were unrelated to SRC effects for pleasant stimuli in both the correlation and the multiple regression analyses. Consequently, the AAT results suggest that the effects of psychopathic traits on approach-avoidance tendencies are indeed limited to cues of threat or danger, rather than showing a general effect on stimulus-incompatible response latencies.

Notably, trait effects on approach-avoidance tendencies toward fear-evoking stimuli were only found for Factor 1 traits, but not for traits captured by the TriPM^16^, in particular not for boldness. Due to its theoretical conception^7^, boldness was assumed to be related to a more positive appraisal of fear-evoking contexts. Recent research, however, failed to link boldness with both dispositional fear enjoyment and subjective appraisal ratings of scary video clips^32^. Although boldness appears to be weakly associated with Factor 1^46^, unlike Factor 1, boldness did not explain variance in SRC effects for fear-evoking stimuli within the present study. Taking both our present observations and previously reported findings^32^ into account, we conclude that fear enjoyment is not represented in TriPM boldness.

Since SRC latency effects are regarded as an indirect measure of the subjective valence of the stimuli presented, some researchers have suggested to use AAT procedures as a diagnostic adjuvant^47^. Based on an elaborate review of the AAT literature, Fricke and Vogel^40^ concluded that specific phobias (but not necessarily other internalizing mental disorders) can be measured reliably with AAT procedures. We argue that, based on the present findings, the AAT might serve a similar purpose in the context of psychopathy. Current self-report measures of psychopathic fearlessness are often heavily laden with excitement/sensation-seeking and impulsivity, making it difficult to determine whether psychopathy is associated with aberrant fear experiences, or with impulsive disinhibition^10^, or both. The AAT allows disentangling behavioral impulsivity from low fear and fear enjoyment, thus providing a potentially useful method to test specific predictions of (developmental) theories surrounding psychopathy. In the present study, the AAT provided further support for the FEH by showing that individuals with elevated psychopathic traits evaluate fear-evoking stimuli as more positive, both when these appraisals were obtained by means of direct (picture valence ratings) and indirect (AAT) assessments.

The present research has a number of advantages, for it is theory-driven, multi-methodological, sufficiently powered, and adheres to open science recommendations (preregistration, open data, and open materials). Nonetheless, our study is not free from limitations. The reliance on self-report data to assess psychopathic traits might be regarded as a particular constraint. In studies that follow the trait approach (i.e., consider psychopathy as a personality trait that is continuously distributed within the general population)—such as the present research -, self-reports are often the only source of information on trait expressions, as objective indicators of antisocial behavior, such as criminal records, are lacking or only available for few participants. In order to evaluate the conceptual traits that are supposedly measured with the scales used in the present study, we conducted factor analyses of the TriPM and SRP 4 items. Parcel-based principal axis factoring supported the notion that the SRP 4 assesses four characteristic personality dimensions, which can be grouped into an interpersonal-affective (PCL/SRP Factor 1) and an antisocial-lifestyle (Factor 2) domain (cf. Supplemental Figure S3). Thus, we conclude that the respective self-reports used herein provide reliable measures of psychopathic traits and behavior. Another potential limitation concerns data acquisition: All data were collected online, including both self-reports and experimental data. An area of concern regarding online data collection is that of fraudulent responding. On the other hand, paper-pencil self-reports are not immune from dishonesty and invalid responding either. Some studies indicate that online surveys may even outperform print questionnaires when it comes to social desirable responding (i.e., they tend to elicit smaller social desirability response bias^48^ or are equally affected at most^49^). The data collected were inspected thoroughly and tested systematically for response bias. Moreover, previous research indicated that the AAT version, which was used in the present study (i.e., the VAAST^45^), is appropriate for online applications^44^. Consequently, we argue that the data quality of our study was overall sufficient. Nevertheless, we acknowledge that the behavior-based assessment used in our study is a relatively recent development. Even though preliminary findings indicate that RT-based measures derived from the VAAST indeed reflect implicit associations and automatic approach-avoidance tendencies^44,45^, the potential of the paradigm to capture inter-individual differences is, so far, relatively unknown. In fact, the relatively small correlation effect sizes obtained in the present study indicate that this potential might be limited. A possible reason for the small magnitude of relationships found between psychopathic traits and AAT response latencies could be the so-called reliability paradox^50^. According to the reliability paradox large experimental effects (i.e., *within-subjects variability*) are typically found when *between-subject variability* is low. Low between-subject variability, however, causes low reliability of the (behavioral) measure in the assessment of individual differences. In line with Rougier et al.^45^ and Aubé et al.^44^, we found large SRC effects (cf. Fig. 2), indicating that our AAT produced robust and large within-subjects effects, which may have had an attenuating impact on the strength of any associations between SRC effects and personality traits. On the other hand, the relatively small associations also could have resulted from relatively low levels of psychopathic traits found in our community-based sample (the mean SRP 4 score was 127 vs. mean SRP scores found in offender samples, e.g. $M = 170$ reported by Tew et al.^51^) or from the quality of the stimulus material in the AAT. Some images of the International Affective Picture System (IAPS) battery seem outdated, and more contemporary standardized stimulus sets were published in recent years. To this end, a replication of our study with updated stimulus sets among selected samples with typically high levels of psychopathic traits, such as violent offender samples, seems warranted.

The present study provides support for a core assumption of the FEH, a theoretical account recently brought forth in order to explain how psychopathic individuals interpret and subjectively experience threatening situations. To this end, the FEH augments previous theories on fear (deficits) in psychopathy, which predominantly focused on the perceptual processing of threat cues^2,25^. With the present study we could replicate and extend recent empirical findings showing that Factor 1 psychopathy traits are linked to more positive appraisals of fear-evoking stimuli^30–32^, given that previous studies assessed this appraisal directly only (i.e., through self-report). By implementing an approach-avoidance-task (AAT), the present study provides the first empirical support for the FEH that stems from indirect assessments of stimulus valence: Factor 1 traits were linked to faster approach towards fear-evoking pictures in the AAT, indicating that these stimuli are perceived as more rewarding (or appetitive, positive) by individuals with elevated interpersonal-affective traits.

Our research highlights the relevance of studies that address the subjective emotional experience of participants in order to obtain conclusive insights on putative emotion deficits in psychopathy. Future research in this field could combine study designs that allow assessing subjective stimulus appraisal and emotional experience in response to (threat) stimuli with physiological measures in order to further our understanding of the neurobiological basis of altered information-processing in psychopathy. Moreover, it would be highly interesting to examine whether the observed psychopathy-related positivity-bias for fear-evoking stimuli is able to predict actual behavioral outcomes, for example with regard to situation selection/decision making, risk taking, or infringement. If this was the case, the appraisal of threatening or fear-inducing cues in psychopathy (as defined by high Factor 1 trait levels) could be connected with etiological considerations from evolutionary psychology (for example Mealy^52^). To this end, future studies should also consider specific samples including, for example, individuals with a criminal record or from correctional settings.

## Methods

### Power considerations

Based on a significant positive correlation between Factor 1 psychopathy traits and affective appraisal scores following fear-provoking video clips ($r=.22$^31^), a-priori estimations of required sample size using G*Power^53^ revealed a minimum sample size of $N = 174$ subjects (one-sided, power = .90, $\alpha =.05$, H0: $r = 0$).

### Sample

Study participants were recruited from the general public in Germany and German-spoken countries (Austria, Switzerland). Data collection was part of a scientific research class in the B.Sc. Psychology program of the host University, which took place in 2019/2020 (online). Of the 321 individuals who initially enrolled in the study, 92 did not complete the full study, whereby the majority discontinued directly before, during, or after the AAT. As preregistered, we excluded data for the following reasons: Eight cases were removed who indicated inattentive or (partly) dishonest study participation at the end of the survey. One subject was excluded due to insufficient language command (less than business fluent), and data of another five participants were excluded due to invariant responding in the Self-Report Psychopathy Scale 4th Edition (SRP 4), the TriPM, and/or the stimulus ratings. In addition, data of four participants were excluded due to comments these subjects provided in a free comments text box at the end of the survey, which suggested potential flawed study participation (e.g., comments indicating difficulties in comprehending the AAT task instructions). The final sample included 211 participants (69% female) with a mean age of 36.55 ± 12.65 years. The sample consisted of an almost equal amount of full-time university students ($n = 75$), part-time students ($n = 66$), and non-students ($n = 70$). Forty-three percent ($n = 90$) reported having completed tertiary education with a formal degree, $n = 89$ individuals reported a high-school diploma/A-Levels equivalent (German Abitur) and $n = 30$ reported school education below that level. Thirty-nine percent of the sample reported being single or divorced; 60% were married or in a serious relationship.

### Design and procedure

The study protocol was approved by the University’s local institutional review board (approval number EA-279-2020) and all procedures were in accordance with the latest version of the Declaration of Helsinki (https://www.wma.net/policies-post/wma-declaration-of-helsinki/). Data were collected online. At the beginning of the study, participants were informed about the study procedure, duration, and technical requirements (e.g., a computer mouse was needed for the experimental part [AAT] of the study). They were further informed about anonymity and the option to discontinue from the study at any time. Upon providing informed consent, participants first completed the self-report scales (see below), which were followed by the experimental procedure (i.e., the AAT). Presentation of stimuli and recording of responses during the AAT were controlled by the software Inquisit (Milliseconds Inc.). Prior to beginning the experiment, participants read instructions and completed practice trials. At the end of the practice, participants received accuracy feedback. During the main task, participants did not receive accuracy feedback.

### Materials and measures

#### Psychopathy measures

*Self-Report Psychopathy Scale, Fourth Edition (SRP 4)* In line with the four-factor model of psychopathy^15^ the 64-item SRP 4^54^ captures four sub-scales with 16 items each that are assigned to two higher-order dimensions: Factor 1, comprising interpersonal-affective psychopathic traits, and Factor 2, which includes antisocial lifestyle features. Respondents are asked to rate their agreement with each statement on a 5-point Likert scale from 1 (*does not apply to me at all*) to 5 (*applies very much*). The scale was validated in both community and forensic samples and shows good indices of internal consistency for the sub-scales representing Factor 1 ($\alpha =.80 -.89$), Factor 2 ($\alpha =.83 -.85$), and the four facets ($\alpha =.71 -.86$, Paulhus et al.^54^). A recent German adaptation of the scale was used^55^. We conducted factor analyses in order to examine and verify the theoretically proposed factor structure of this version (for further details, see below, Data Preprocessing and Statistical Analyses section).

*Triarchic Psychopathy Measure (TriPM)* The TriPM^16^ comprises 58 items which were developed to capture the three trait dimensions meanness, boldness, and disinhibition. Responses are provided on a 4-point Likert scale from 1 (*incorrect*) to 4 (*correct*). Even though the internal consistency coefficients reported for the three sub-scales are good ($\alpha =.89 -.99$^56^), the three-dimensional structure of the TriPM has been questioned^57,58^ and is a matter of ongoing debate^46,59^. We used the German translation provided by Eisenbarth et al.^60^ in our study and, as for the SRP 4 items, conducted factor analyses to test the appropriateness of its expected factor structure (see Data Preprocessing and Statistical Analyses).

#### Approach-avoidance tendencies

**Fig. 1:**
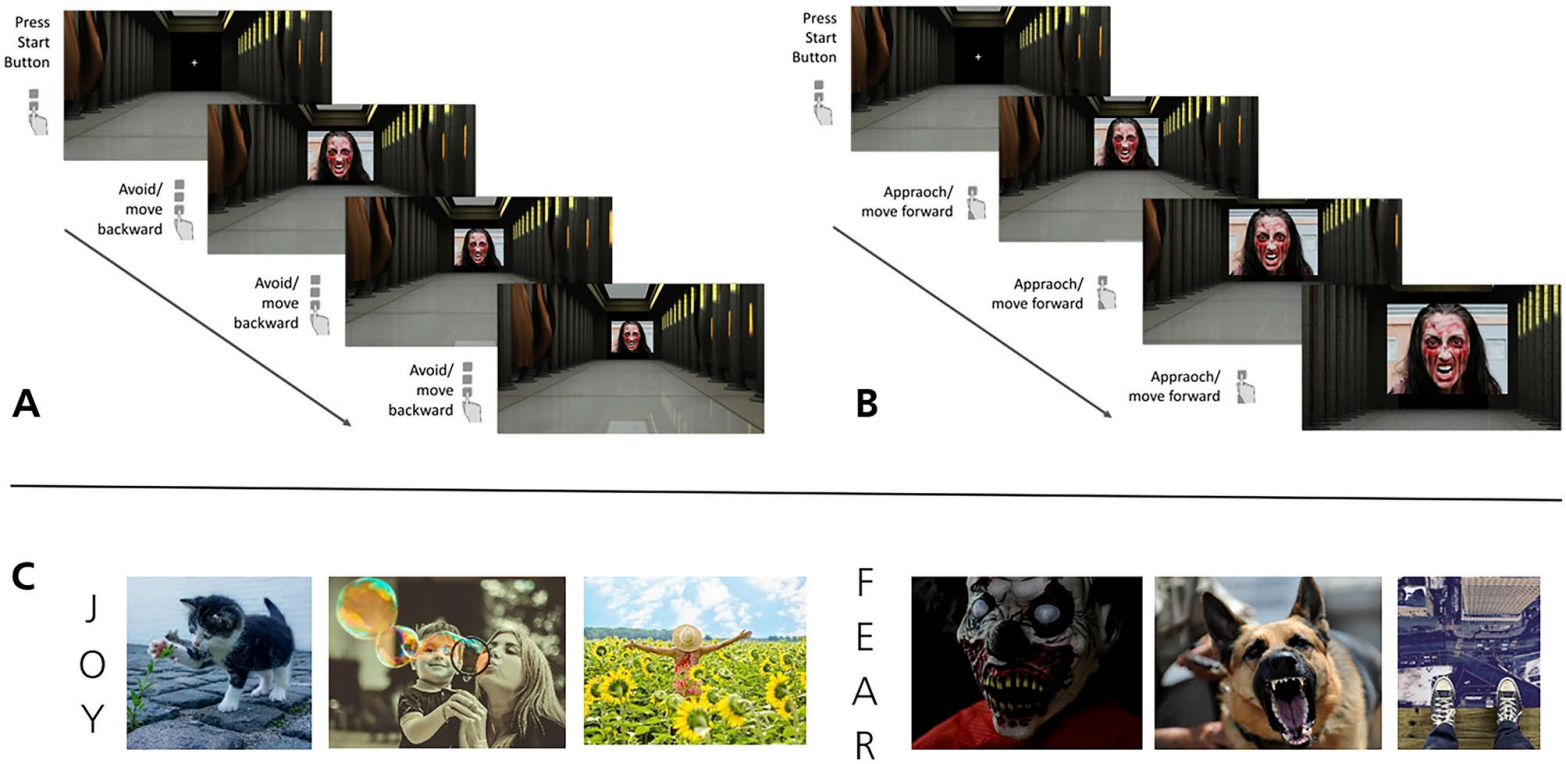
Illustration of the approach-avoidance task design used in the present study. (**A**) Stimulus-response-compatible task block (avoid fear-evoking, approach joy images); (**B**) Stimulus-response-incompatible task block (avoid joy, approach fear-evoking images). (**C**) Example stimuli of joy (left) and fear-evoking (right) picture stimuli used.

We assessed automatic approach and avoidance tendencies with a picture version of the Visual Approach-Avoidance of the Self Task (VAAST)^45^, which has been tested to be suitable for online applications^44^. The VAAST presents stimuli of different categories (here: pleasant/joyful and fear-evoking/threat images) within a three-dimensional environment on screen and, depending on the instructions provided within the respective task block, participants press designated response buttons to virtually approach the stimuli of one category while virtually avoiding the other. When the ’approach’-key is used, the stimulus and its surrounding 3D environment are gradually enlarged, creating the impression of the participant moving towards the stimulus (see Fig. 1). When the ’avoidance’ key is pressed, in turn, stimulus and environment become gradually smaller, creating the visual impression of the person moving away or backward. Like most AATs, the VAAST consists of two task blocks, an SR-compatible block (Fig. 1A), in which subjects are instructed to avoid (i.e., press the ’avoid’-key and move away from) fear-evoking images and approach (use the ’approach’-key in order to move towards) pleasant images; and an SR-incompatible block (Fig. 1B), in which subjects are required to avoid pleasant images and approach fear-evoking ones. To start a new trial (i.e., the presentation of a new picture stimulus on screen), participants press a start button on the keyboard and are instructed to respond as quickly as possible after stimulus-onset. Due to the two stimulus categories used in the present study, the VAAST included the following four conditions: (1) approach-pleasant, (2) avoid-threat (both SR-compatible), (3) approach-threat, (4) avoid-pleasant (both SR-incompatible). A set of 15 pictures per stimulus category were included, whereby each was presented twice per task block, resulting in 60 SR-compatible (30 pleasant, 30 fear-evoking) and 60 SR-incompatible (again, 30 pleasant, 30 fear-evoking) VAAST trials. Six additional trials per block with pictures that were not included in the main task blocks were used as practice trails. Participants could take a self-timed break between task blocks. The pictures used in the VAAST were taken from the International Affective Picture System (IAPS)^61^ and were selected based on a pilot study, in which $N = 20$ (50 % male) volunteers rated 87 IAPS pictures, 48 of which were taken from the IAPS categories ’threatening’ (’human threat’ and ’animal threat’) and 49 images from the category ’pleasant’. In the pilot study, the images were rated with respect to their valence and arousal levels on a Likert-type response scale ranging from -3 to +3 (valence) and from 1 to 7 (arousal), respectively. Based on these ratings, 15 final images per category were chosen which discriminated best between the two stimulus categories with respect to valence (threat images: $M = -2.0, SD =.059$; pleasant images: $M = 1.7, SD = 0.35$), while affording relatively similar arousal ratings (threat images: $M = 4.70, SD = 0.50$; pleasant images: $M = 3.86, SD = 0.46$). In this way distinct responses to threatening and pleasant stimuli could be ascribed to differences in their affective content rather than being a consequence of divergent arousal levels. Example stimuli and code used to program the VAAST for the Inquisit software (Millisecond Inc.) are provided at https://osf.io/7sxtw/.

#### Subjective stimulus ratings

Upon completing the AAT, all participants were presented with the 30 picture stimuli (15 pleasant, 15 fear-evoking) once again. They were asked to rate the picture stimuli with respect to their overall subjective valence on a 7-point Likert-type scale from 1 (*very negative*) to 7 (*very positive*).

### Data preprocessing and statistical analyses

In line with joystick-based AAT studies^62^, we calculated median RTs for all trials belonging to the same VAAST condition. In addition, we computed SRC effects by subtracting median RTs (for either threat or pleasant stimuli, respectively) obtained in the compatible condition from median RTs obtained in the incompatible condition. Thus, positive SRC effects denote enhanced RTs in incompatible relative to compatible conditions. As RTs obtained in AAT experiments are usually positively skewed, we applied an outlier cut-off to normalize the RT data distribution and to exclude overly fast and overly slow responses in the VAAST (below 200 ms/above 5000 ms, these cut-offs were pre-registered and are based on Klein et al.^47^) from further analyses. Participants were further excluded if reaction patterns showed signs of distraction (i.e., more than 30% incorrect responses per task block). Moreover, we excluded cases who showed signs of invalid response patterns in one of the psychopathy measures (as reflected by zero variance in item responses for the SRP 4 or TriPM) or if they reported insufficient language proficiency (at least fluent German was required).

Psychopathy trait scores were obtained by calculating mean scale scores of the SRP 4 and TriPM as described within the respective manuals^16,54^. In order to examine the validity of the respective item-to-scale assignments we conducted a series of factor analyses using R (version 4.3.2)^63^ and report the results within the Supplemental Material of this article. First, we conducted exploratory factor analyses (EFA) with principal axis factoring and an oblique rotation method (promax) of the SRP 4 and TriPM items, respectively. Bartlett’s test of sphericity was significant and the Kaiser-Meyer-Olkin (KMO) criterion also indicated data suitability for EFA for both the TriPM ($\chi ^2(1635) = 5224.65$, $p <.001$; KMO $=.79$) and the SRP 4 ($\chi ^2(1635) = 5689.37$, $p <.001$; KMO $=.78$). With respect to the TriPM, both the scree plot (see Supplemental Figure S1) and the Hull method indicated three factors, whereas parallel analysis (PA) suggested seven and the Kaiser-Guttman Criterion (KGC) six factors to extract. The pattern matrix of the rotated three-factor solution for the TriPM items is shown in Supplemental Table S1. It shows that the EFA-based item-factor assignment was well in accordance with the intended TriPM structure with respect to the factors boldness and disinhibition, and somewhat less (68% agreement) for the meanness factor. For the SRP 4, results concerning the factors to retain varied substantially between methods: While the scree plot (see Supplemental Figure S2) suggested four factors, the Hull method suggested one factor, PA indicated 10, and KGC seven factors to retain. The pattern matrix of the rotated four-factor solution (in accordance with scree plot and theory) for the SRP 4 items is shown in Supplemental Table S2. Of note, it can be assumed that the present sample size was too small for robust EFA, as the sample-to-item ratio should not be less than 5-1^64^.With regard to the 64 items of the SRP 4, for example, a minimum sample size of 320 participants would be required according to this guideline. Together with these sample size restrictions, limitations of the EFA approach to appropriately model hierarchical factor structures (which has been proposed for the SRP 4^54^) may have led to the discrepant results concerning the number of factors to retain and probably caused distorted parameter estimates (i.e., EFA-based factor loadings). To overcome this issue, we further conducted parcel-based confirmatory factor analyses with robust maximum likelihood (MLR) parameter estimation for the SRP 4, as suggested by different sources^54,65^. The results of these final structural analyses are displayed in Supplemental Figure S3 and indicated acceptable fit for both the correlated four-factor model (Comparative Fit Index [CFI] $=.91$; Root Mean Square Error of Approximation[RMSEA] $=.071$) and the hierarchical four-factor model with two higher-order dimensions representing PCL Factor 1 and Factor 2 (CFI $=.91$; RMSEA $=.072$). Factor loadings for parcels and the inter-factor correlations align with SRP 4 theory^54^ and previous confirmatory analyses of the scale’s latent structure^65^, see Supplemental Figure S3.

We conducted bivariate correlation-analyses to test the hypotheses (see below; the online preregistration can further be retrieved from https://osf.io/7sxtw/). We hypothesized that higher expressions of Factor 1 psychopathic traits (Hypothesis 1a) and boldness (Hypothesis 1b) are associated with faster approach towards fear-evoking pictures (i.e., a negative correlation between psychopathic trait measures and median RTs to fear-evoking stimuli in the SR-incompatible AAT condition). With respect to the SR-compatible AAT condition, we assumed that higher expressions of Factor 1 traits (Hypothesis 2a) and boldness (Hypothesis 2b) are associated with slower avoidance of fear-evoking pictures (i.e., a positive correlation between trait measures and RTs to fear-evoking pictures in the SR-compatible condition). As a difference measure, the SRC effect depicts a stimulus-response bias as a function of the approach-fear versus avoid-fear condition. Since fear-evoking cues are typically considered as aversive or negative, the generally expected SRC effect for this stimulus category would reflect faster RTs during their avoidance, as compared to their approach. If psychopathic traits, as predicted by the FEH, correspond with more positive appraisal of threat, the SRC effect for fear-evoking pictures should decrease with elevated psychopathic traits. Hence, we further expected to find a negative correlation between the SRC effect for fear-evoking stimuli and Factor 1 psychopathic traits (Hypothesis 3a) and boldness (Hypothesis 3b), respectively. We conducted one-sided significance tests ($\alpha =.01$) for all correlation coefficients.

In addition to the bivariate correlation analyses, we performed multiple regression analyses in order to test whether interpersonal-affective psychopathic traits (Factor 1 within the two-factor model) or boldness (from the triarchic model) incrementally explained variance in SRC effects for fear-evoking stimuli. To this end, two step-wise regression models—one per psychopathy model (two-factor model and triarchic model, respectively)—were tested, with fear-related SRC effects as the dependent variable. Thus, the first regression model included Factor 1 and Factor 2 (added at step 2) as predictors, the second regression model involved boldness (step 1), meanness, and disinhibition (step 2) as predictors.

Eventually, we conducted correlation analyses to examine associations between psychopathic traits (again: Factor 1, boldness), and the post-experimental stimulus valence ratings provided by all study participants subsequent to the AAT.

**Fig. 2:**
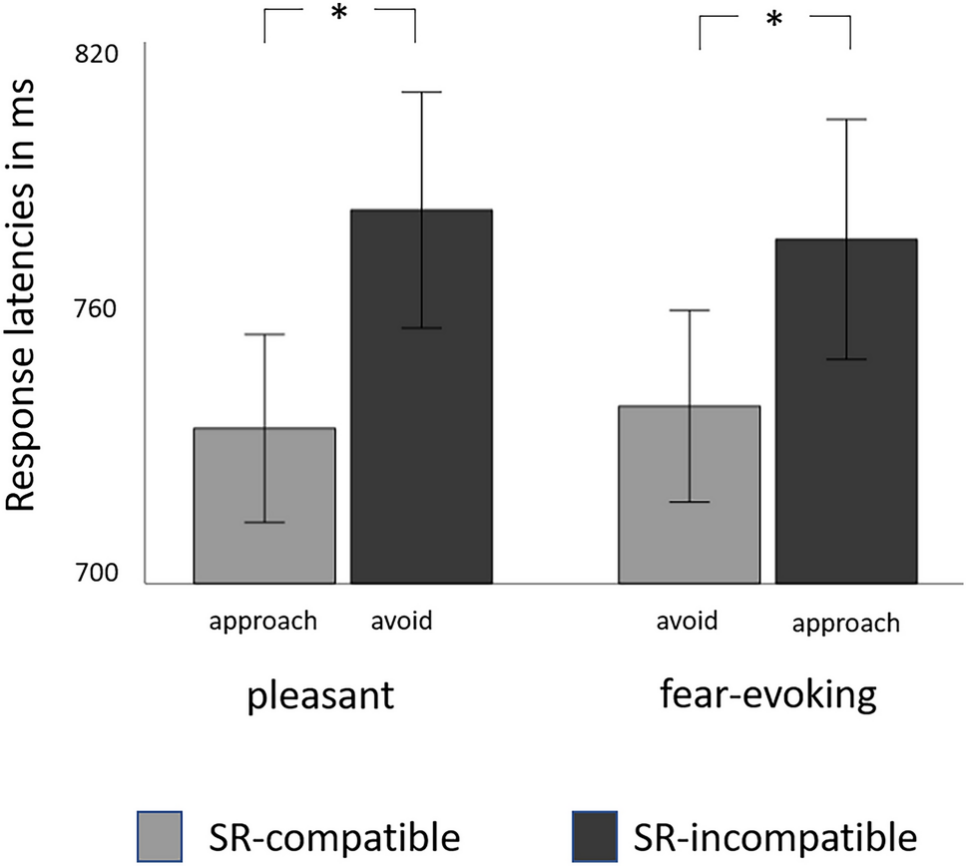
Illustration of the stimulus-response compatibility effect in the Approach-Avoidance Task (AAT). AAT approach-avoidance task. Error bars represent $\pm 2$ standard errors. * $p <.001$.

**Table 1:** Descriptive statistics and bivariate interrelations of study variables. (1) to (6)—subscales/dimensions of the SRP 4; (7) to (9)—subscales/dimensions of the TriPM; (10)–(13)—Median reaction times (RT) obtained within the respective AAT task condition; (14)–(15)—mean subjective valence ratings (from very negative to very positive) of picture stimuli used in the AAT. $\alpha$ indicates internal consistency (Cronbach’s $\alpha$ for self-report scales, split-half Spearman-Brown coefficient for AAT reaction times). Coefficients with $p <.01$ are displayed in bold.

Item	*M*	*SD*	$\alpha$	(1)	(2)	(3)	(4)	(5)	(6)	(7)	(8)	(9)	(10)	(11)	(12)	(13)	(14)	(15)
(1) Factor 1	68.26	15.92	.87	—														
(2) Interpersonal	35.35	9.33	.82	**.91**	—													
(3) Affective	32.91	8.36	.77	**.89**	**.62**	—												
(4) Factor 2	59.08	13.26	.81	**.58**	**.52**	**.53**	—											
(5) Antisocial	21.72	6.59	.71	**.46**	**.38**	**.45**	**.78**	—										
(6) Lifestyle	37.36	9.13	.77	**.52**	**.48**	**.45**	**.89**	**.41**	—									
(7) Boldness	2.64	0.40	.81	**.27**	**.20**	**.30**	**.33**	**.22**	**.32**	—								
(8) Meanness	1.53	0.37	.86	**.67**	**.55**	**.66**	**.53**	**.40**	**.49**	**.19**	—							
(9) Disinhibition	1.62	0.36	.85	**.34**	**.36**	**.24**	**.60**	**.38**	**.60**	**-.20**	**.44**	—						
(10) Approach Fear	778	196	.84	**−.20**	**−.18**	**−.18**	−.13	−.08	−.13	−.05	−.08	−.12	—					
(11) Approach Joy	738	154	.93	−.14	−.14	−.11	−.09	−.00	−.13	.04	−.05	−.13	**.76**	—				
(12) Avoid Fear	742	156	.91	−.11	−.12	−.08	−.11	−.05	−.13	.03	−.03	−.13	**.79**	**.87**	—			
(13) Avoid Joy	787	194	.86	**−.22**	**−.17**	**−.22**	**−.17**	−.12	−.16	−.02	−.13	**−.17**	**.93**	**.75**	**.80**	—		
(14) ratings joy	5.89	0.61	.91	**−.23**	**−.24**	−.16	−.12	−.03	−.15	−.06	−.15	**−.20**	.06	.02	−.02	.06	—	
(15) ratings fear	1.78	0.55	.89	**.37**	**.32**	**.34**	**.30**	**.27**	**.25**	−.00	**.28**	**.20**	−.03	−.07	.02	−.10	**−.46**	—

**Table 2:** Results of multiple regression analyses of SRC effects with psychopathic traits as dependent variables. Bootstrapped (1000 samples) coefficients (b) and standard errors (SE) were used. 95% confidence intervals (CI) were determined based on the bias corrected and accelerated (BCA) method. fear – SRC effects for fear-evoking stimuli served as dependent variable ($R^2 =.034; F(2,201) = 3.57, p =.03$); joy – SRC effects for joy stimuli served as dependent variable ($R^2 =.013; F(2,199) = 2.29, p =.10$).

	*b*	*SE*	95%-CI	$\beta$	*p*
Fear					
Factor 1	−1.62	0.65	[−2.97;−0.19]	−0.21	.01
Factor 2	.53	0.72	[−0.76;1.80]	0.06	.47
Joy					
Factor 1	−0.77	0.64	[−2.02;0.44]	−0.10	.24
Factor 2	−0.74	0.76	[−2.25;0.70]	−0.07	.33

## Supplementary Information

Supplementary Information.

## Data Availability

The study pre-registration, materials (experimental code), data, analysis code, and any supplemental materials are available at https://osf.io/7sxtw/files/osfstorage.
