# Supplementary material for: Psychopathic personality traits are associated with experimentally induced approach and appraisal of fear-evoking stimuli indicating fear enjoyment
Source: Sci Rep. 2025 Mar 13;15:8646. doi: 10.1038/s41598-025-91652-2 (PMC11906771; doi:10.1038/s41598-025-91652-2)
Supplement: Supplementary file 1 — Supplementary Information. [file 41598_2025_91652_MOESM1_ESM.pdf]

Supplementary Materials

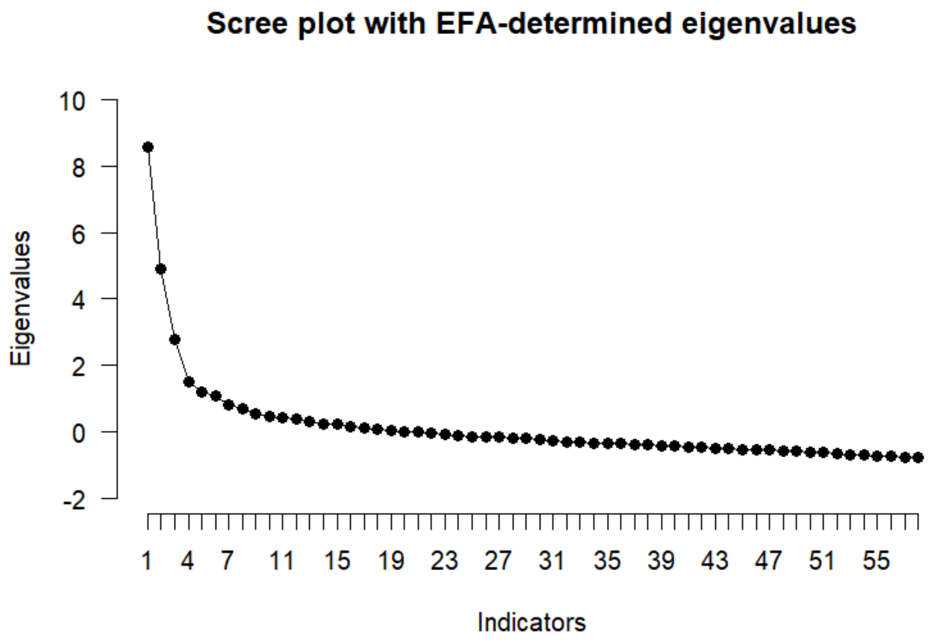

Figure S1. Scree Plot for the 58 Items of the TriPM.

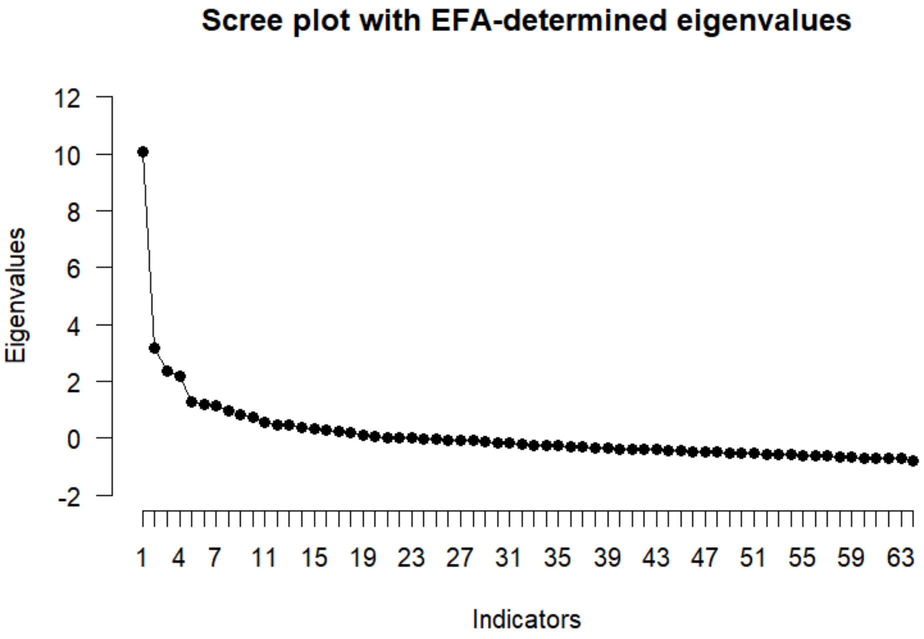

Figure S2. Scree Plot for the 64 Items of the SRP 4.

Item	Factor 1 Disinhibition	Factor 2 Meanness	Factor 3 Boldness
1.	-.313	.018	**.266**
2.	-.211	**.734**	-.119
3.	**.293**	-.144	.076
4.	.023	-.080	**.287**
5.	**.460**	-.046	.024
6.	.140	.111*	*.458*
7.	-.543	.079	**.355**
8.	.028	**.532**	-.117
9.	**.624**	-.105	.058
10.	-.508	.195	**.341**
11.	-.117	**.730**	-.185
12.	**.245**	.217	-.122
13.	.012	-.132	**.702**
14.	.120	.131*	*.485*
15.	**.600**	-.140	.332
16.	-.460	-.002	.089*
17.	*.256*	.226*	.025
18.	**.478**	-.095	.068
19.	.210	-.237	**.634**
20.	-.066	**.620**	.076
21.	**.589**	.008	-.195
22.	-.336	-.066	**.581**
23.	.293	**.309**	.183
24.	**.370**	-.032	.131
25.	-.308	.048	**.115**
26.	*.302*	.294*	.117
27.	**.557**	-.157	-.030
28.	-.193	.153	**.476**
29.	-.020	**.661**	-.041
30.	**.406**	.145	-.090
31.	**.621**	.017	.165
32.	-.008	.044	**.384**
33.	-.102	**.703**	-.054
34.	.314*	*.344*	.116
35.	-.386	.037	**.447**
36.	-.069	**.808**	-.036
37.	**.597**	.087	.104
38.	.099	-.071	**.567**
39.	*.195*	**.195**	-.082
40.	*.324*	.330*	.111
41.	-.072	-.038	**.539**
42.	.262	**.414**	.116
43.	**.337**	-.005	.078
44.	-.486	.271	**.364**
45.	.267*	-.020	*.480*

Table S1. Pattern Matrix Resulting from Exploratory Factor Analysis of the TriPM Items.
Principal axis factoring with promax rotation was used. Bold numbers indicate primary factor loadings for the respective item that are in accordance with the scale's theory/manual. Italic numbers indicate theory-deviant primary loadings, whereby numbers with asterisk (*) show the theory-derived factor assignment of the respective item. Factor loadings $< .10$ are not highlighted.

Item	Factor 1<br>Disinhibition	Factor 2<br>Meanness	Factor 3<br>Boldness
46.	**.542**	-.195	.097
47.	.024	.063	**.330**
48.	.098	**.731**	-.037
49.	**.363**	.297	-.123
50.	-.423	-.063	**.366**
51.	**.479**	.079	-.054
52.	-.076	**.658**	-.091
53.	**.321**	.231	.061
54.	-.021	.096	**.123**
55.	-.103	**.638**	.071
56.	**.303**	.200	.056
57.	.131	-.213	**.397**
58.	**.185**	.119	.113

Table S1. Pattern Matrix Resulting from Exploratory Factor Analysis of the TriPM Items (*cont.*).
Principal axis factoring with promax rotation was used. Bold numbers indicate primary factor loadings for the respective item that are in accordance with the scale's theory/manual. Italic numbers indicate theory-deviant primary loadings, whereby numbers with asterisk (*) show the theory-derived factor assignment of the respective item. Factor loadings $< .10$ are not highlighted.

Item	Factor 1<br>INT	Factor 2<br>AFF	Factor 3<br>ANTI	Factor 4<br>LIFE
SRP1	.109	-.182	-.089	**.534**
SRP2	-.046	.153*	-.120	*.579*
SRP3	.215*	*.298*	-.033	.137
SRP4	-.017	-.217	.042	**.432**
SRP5	-.239	.185	.100*	*.271*
SRP6	-.243	.118	.128*	*.205*
SRP7	*.567*	.107*	-.076	-.123
SRP8	**.642**	-.050	-.180	.043
SRP9	.171	.065	-.252	**.611**
SRP10	.144	.032	.188*	*.243*
SRP11	-.099	**.288**	.157	.021
SRP12	-.088	.025	**.717**	.023
SRP13	**.321**	.054	*.321*	.001
SRP14	.027	-.104	.005	**.202**
SRP15	.052	.237*	.038	*.242*
SRP16	**.309**	.172	.023	.117
SRP17	-.178	.266	-.169	**.582**
SRP18	.056	.003	**.371**	.036
SRP19	.030	**.482**	.172	-.138
SRP20	**.501**	.135	-.110	.181
SRP21	-.077	.183	**.206**	.180
SRP22	.067	-.237	*.288*	.136*
SRP23	*.171*	.136*	-.020	.058
SRP24	.200*	*.270*	.103	-.163
SRP25	.015	.246	-.160	**.291**
SRP26	.112	**.506**	.052	-.021
SRP27	**.471**	.058	-.042	.173
SRP28	.020	-.082	-.148	**.701**
SRP29	-.086	.140	**.297**	.248
SRP30	-.037	-.001*	*.176*	.021
SRP31	.245*	.042	-.104	*.298*
SRP32	.041	-.062	.141	**.489**
SRP33	.023	**.586**	.055	.024
SRP34	-.232	.199	**.271**	.204
SRP35	**.445**	.214	.254	-.134
SRP36	.056	.151	-.074	**.300**
SRP37	.332	**.369**	.052	-.046
SRP38	.066*	*.349*	-.277	.213
SRP39	.116	-.030	.138	**.291**
SRP40	.188	**.267**	-.003	.259
SRP41	**.668**	.076	-.114	-.049
SRP42	.157	-.321	.141	**.282**
SRP43	.003	-.104	**.315**	.198
SRP44	-.104	**.707**	-.143	.228
SRP45	**.272**	.090	-.014	.197

Table S2. Pattern Matrix Resulting from Exploratory Factor Analysis of the SRP 4 Items.
Principal axis factoring with promax rotation was used. Bold numbers indicate primary factor loadings for the respective item that are in accordance with the scale's theory/manual. Italic numbers indicate theory-deviant primary loadings, whereby numbers with asterisk (*) show the theory-derived factor assignment of the respective item.
INT – Interpersonal; AFF – Affective; ANTI – Antisocial; LIFE – Lifestyle.

Item	Factor 1 INT	Factor 2 AFF	Factor 3 ANTI	Factor 4 LIFE
SRP46	.020	-.005	-.004*	*.261*
SRP47	-.324	.245	-.194	**.720**
SRP48	*.497*	.004*	.018	-.057
SRP49	.098	.048	**.596**	-.200
SRP50	**.678**	-.091	-.177	-.088
SRP51	*.429*	-.371	.225	.084*
SRP52	.059	.102	**.514**	-.058
SRP53	.128	**.449**	.204	-.105
SRP54	**.794**	-.053	-.200	-.126
SRP55	*.492*	-.151	-.048	.180*
SRP56	.006	**.363**	.297	-.109
SRP57	-.089	-.040	**.719**	-.068
SRP58	**.562**	.248	.044	-.257
SRP59	*.411*	-.136	.163	.137*
SRP60	*.565*	.017*	.095	.076
SRP61	**.299**	.088	-.088	.125
SRP62	-.050	-.111	**.442**	.135
SRP63	-.135	.038	**1.043**	-.230
SRP64	-.123	.024	**.889**	-.217

Table S2. Pattern Matrix Resulting from Exploratory Factor Analysis of the SRP 4 Items (*cont.*).
Principal axis factoring with promax rotation was used. Bold numbers indicate primary factor loadings for the respective item that are in accordance with the scale's theory/manual. Italic numbers indicate theory-deviant primary loadings, whereby numbers with asterisk (*) show the theory-derived factor assignment of the respective item.
INT – Interpersonal; AFF – Affective; ANTI – Antisocial; LIFE – Lifestyle.

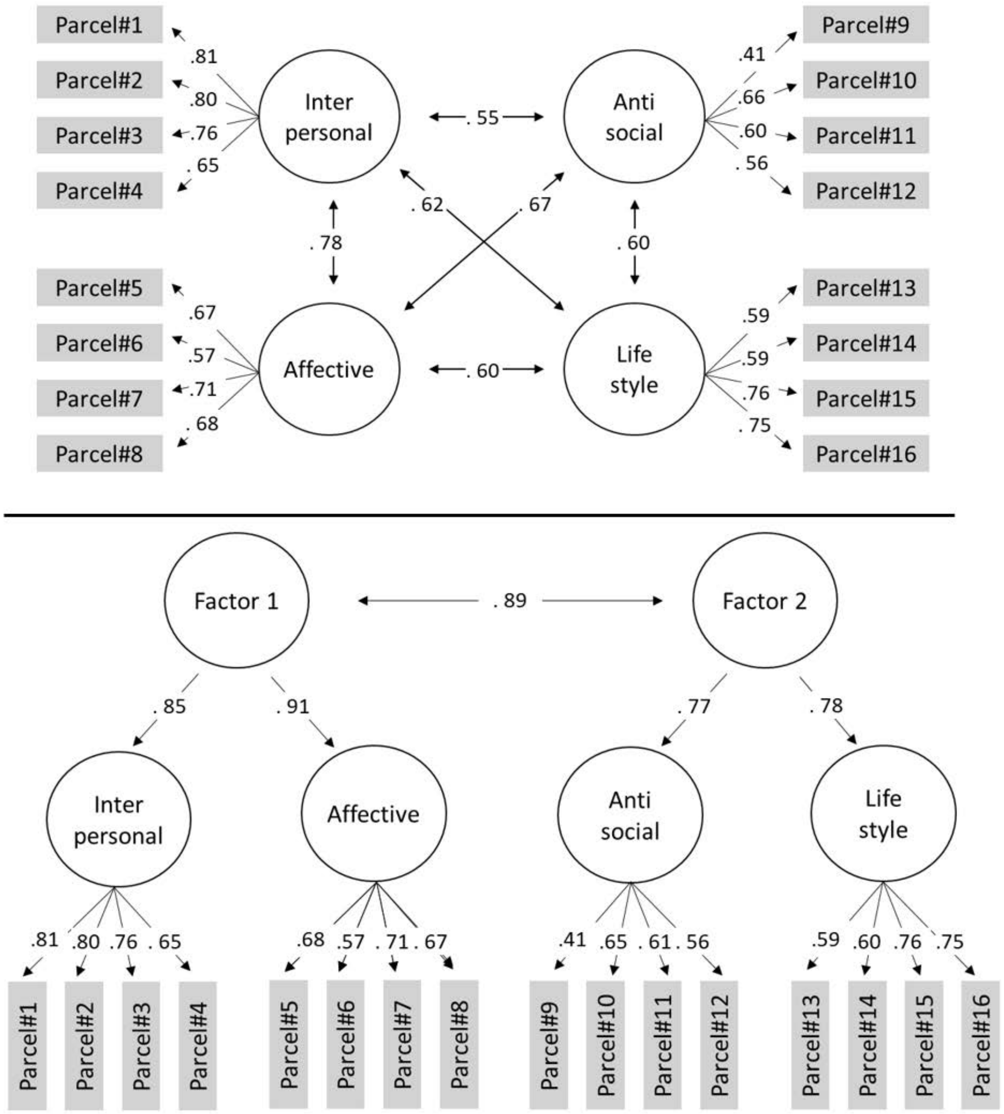

Figure S3. Parcel-based Correlated Four-Factor Model (Upper Panel) and Hierarchical Factor Model (Lower Panel) of the SRP 4.
Both models showed almost identical and acceptable fit (CFI = .91; RMSEA = .07. Table S3 displays item allocations in parcels.)

Item	Parcel	Item	Parcel	Item	Parcel	Item	Parcel
SRP3	1	SRP2	5	SRP5	9	SRP1	13
SRP8	1	SRP7	5	SRP6	9	SRP4	13
SRP13	1	SRP11	5	SRP10	9	SRP9	13
SRP18	1	SRP15	5	SRP12	9	SRP14	13
SRP20	2	SRP19	6	SRP18	10	SRP17	14
SRP24	2	SRP23	6	SRP21	10	SRP22	14
SRP27	2	SRP26	6	SRP29	10	SRP25	14
SRP31	2	SRP30	6	SRP34	10	SRP28	14
SRP35	3	SRP33	7	SRP43	11	SRP32	15
SRP38	3	SRP37	7	SRP46	11	SRP36	15
SRP41	3	SRP40	7	SRP49	11	SRP39	15
SRP45	3	SRP44	7	SRP52	11	SRP42	15
SRP50	4	SRP48	8	SRP57	12	SRP47	16
SRP54	4	SRP53	8	SRP62	12	SRP51	16
SRP58	4	SRP56	8	SRP63	12	SRP55	16
SRP61	4	SRP60	8	SRP64	12	SRP59	16

Table S3. Item-Parcel Assignments Used in the Confirmatory Factor Analyses of the SRP 4. Item wordings can be retrieved from the SRP 4 manual[54].
